# The GAIRS Checklist: a useful global assessment tool in patients with Rett syndrome

**DOI:** 10.1186/s13023-022-02259-z

**Published:** 2022-03-05

**Authors:** Rosa Angela Fabio, Martina Semino, Samantha Giannatiempo

**Affiliations:** 1 Department of Clinical and Experimental Medicine, via Bivona, 98121 Messina, Italy; 2 CARI, (Airett Center Innovation and Research), Vicolo Volto S. Luca, 16, 37100 Verona, Italy; 3 Catholic University of Milan, Largo Gemelli, 1, 20100 Milan, Italy

**Keywords:** Rett syndrome (RTT), Assessment, Global function, GAIRS Checklist

## Abstract

**Background:**

Rett Syndrome is a severe, neurodevelopmental disorder mainly caused by mutations in the MECP2 gene, affecting around 1 in 10,000 female births. Severe physical, language, and social impairments impose a wide range of limitations in the assessment of the abilities of Rett patients. This study proposes an analysis and first validation of a Global Assessment and Intervention in Rett syndrome (GAIRS) Checklist for assessing behavioral, intellectual, academic, neuropsychological and psychosocial manifestations in patients with Rett Syndrome. We administered the GAIRS Checklist to 113 Italian patients with Rett Syndrome aged 4–42.

**Aims of this study:**

To examine the psychometric characteristics of the GAIRS Checklist. Moreover, the aim is also to examine the validity of GAIRS with test–retest correlation, convergent validity with similar functional measurements, such as the Vineland scales, and divergent validity with severity of disease scale, such as the RARS scale and severity of neuropsychiatric evaluations.

**Results:**

All 10 subscales of GAIRS were positively and significantly related to each other and to the total GAIRS score, and the subscales showed high levels of Cronbach’s alpha values (from .77 to .95). Principal axis factoring suggested two factors that explain 60% of the variance. Test–retest reliability is 0.82. This means that psychometric properties are reliable. Correlation for Concurrent validity with Vineland score was high and Divergent Validity with RARS was also high.

**Conclusion:**

The GAIRS Checklist used for Rett syndrome is acceptable and feasible to complete assessment in a clinical setting. Moreover, it can detect the complexity of this disease and may suggest the next step in terms of specific training in Rett syndrome.

## Background

Rett syndrome (RTT) is a severe, neurodevelopmental disorder mainly caused by mutations in the MECP2 gene, affecting around 1 in 10,000 female births [1]. Clinical manifestations include severe linguistic and motor impairments that are the core of phenotype symptoms. A child affected by RTT initially appears to follow a typical development path, but at about 18 months of age a subtle regression in developmental acquisitions begins, opening the path to the clinical stages [2]. Loss of previously acquired language skills and of purposeful hand use, increasing difficulties in motor abilities (dyspraxia) and mental retardation are the clearest signs of regression involved in RTT. Other typical signs of RTT appear including hand stereotypies—such as handwashing, hand-wringing, hand-mouthing—breathing disorders (breath holding and hyperventilation), ataxia, agnosia, bruxism and epilepsy [3–6].

Several assessments on patients with RTT have been conducted on cognitive abilities, on communicational abilities and on motor abilities [7–11]; only few of these have carried out a global functional assessment of all the abilities of these patients. Assessment of the cognitive skills of patients with RTT, as well as other individuals with severe motor and communication limitations, is extremely challenging. [7] Neuropsychological and cognitive assessments are generally developed for and standardized with typically developing children who do not have physical impairments [12]. When standard assessment procedures requiring manual motor functioning for responding have been used to evaluate cognitive functioning, patients with RTT generally achieve age-equivalent performances close to young infants [13, 14]. Severe physical, language, and social impairments impose a wide range of limitations [2–5]. With reference to communication abilities, some studies [15, 16] have aimed to recognize any communicative acts (vocalizations, hand movement stereotypies, body movements, facial expressions, or eye gaze) that would represent a consistent and unequivocal response used by participants with RTT.

With reference to the assessment of motor abilities, available clinical scales do not comprehensively capture the variability of presentation of gait and postural abnormalities in Rett Syndrome patients. Griffiths scales [17] appear too specific to assess peculiar patterns of (loco-)motor derangements in RTT. In addition, the standardization sample included children aged 0–72 months and therefore, similar to the standardization sample of the Bayley scales [18], is not appropriate to test older children or adolescents. There is another motor scale specific for RTT and already validated: the Rett Syndrome Gross Motor Scale (RSGMS) [19]. Rodocanachi et al. [20], in a new standardized scale, the Rett Syndrome Motor Evaluation Scale (RESMES), added postural transitions and walking up or downstairs. RESMES items are centered on the International Classification of Functioning construct of patient’s capacity, which reflects what an individual can do in a semi-standardized environment and have been conceived to capture fine-grained characteristics of movement. Regarding a general assessment, there are two scales that can be used to assess children with special needs: the Portage Guide to Early Education Checklist [21] and the Bayley Scales of Infant Development [18]. The Portage Guide to Early Education Checklist [21] was developed by the Portage Project home intervention program which has been serving preschool multiple children with special needs in Wisconsin, USA. The guide has two parts: a developmental checklist which lists sequential behaviors from birth through five years of age in five learning areas and a set of curriculum cards which match each of the 450 behaviors listed on the checklist. The check-list has been used to pinpoint behaviors and measure change. The cards have been used in establishing individual home training prescriptions [21]. The Bayley Scales of Infant Development [18] consist of a mental scale of 103 items and a motor scale of 81 items. The scales have been designed to measure growth and development from birth to 30 months of age. The instrument was developed primarily for clinical and research use. According to Holden [18], the Bayley Scales have been invaluable in filling a longstanding need for a well standardized, reliable instrument to assess the developmental progress of infants.

With reference to the global assessment of the abilities in Rett syndrome, there are few instruments that can measures the global functional abilities of RTT patients. Some assessments have been carried out by interviewing the parents or caregivers using the Vineland Adaptive Behavior Scales-Interview second edition (VABS), and the Rett Assessment Rating Scales (RARS) [22]. VABS [23] is subdivided into four domains: communication; daily living; socialization; and motor skills. The interviewer asks general questions pertaining to the patient’s functioning in each domain and uses the responses to rate the examinee on each critical behavior item (2: always present, 1: sometimes present, 0: seldom or never present). Typical interviews require approximately one hour. A total score is computed by summing the individual ratings for each scale. Another general assessment scale has been created to evaluate level of severity in RTT patients—RARS [22]. This scale is organized into seven domains: cognitive, sensorial, motor, emotional, autonomy, typical characteristics of the disease and of behavior. A total of 31 items was generated as representative of the profile of RTT. Each item is provided with a brief glossary explaining its meaning in a few words. Each item is rated on a 4-point scale, where 1 = within normal limits, 2 = infrequent or low abnormality, 3 = frequent or medium–high abnormality, and 4 = strong abnormality. Intermediate ratings are possible; for example, an answer between 2 and 3 points is rated as 2.5. For each item, the evaluator circles the number corresponding to the best description of the patient. After a patient has been rated on all 31 items, a total score is computed by summing the individual ratings. This total score allows the evaluator to identify the level of severity of RTT, conceptualized as a continuum ranging from mild symptoms to severe deficits (Mild = 0–55; Moderate = 56–81; Severe =  > 81).

Summarizing, the main limitations of the scales presented here are firstly, in some scales like VABS and RARS parents and caregivers are requested to evaluate patients’ abilities through simple observation and not through direct evaluation by therapists; secondly, in some of these scales not all the areas of manifestations in RTT are considered (behavioral, intellectual, academic, neuropsychological and psychosocial); finally not all of these scales are presented hierarchically and they may not be valid for the assessment or for suggesting all the next level of the steps necessary for any eventual intervention.

In the present work, we proposed the Global Assessment and Intervention in Rett syndrome (GAIRS) to overcome all these limitations. The aim of this study is to examine the preliminary psychometric characteristics of the GAIRS Checklist. Furthermore, the aim is to examine the validity of GAIRS with test–retest correlation, convergent validity with similar functional measurements such as the Vineland scales, divergent validity with severity of disease scale such as the RARS scale and neuropsychiatric evaluations. A specific GAIRS Checklist has been developed to assess behavioral, intellectual, academic, neuropsychological and psychosocial manifestations in patients with Rett Syndrome. The purpose of the Checklist is to offer an easy-to-use, short and accessible tool for every health-care professional to assess all the abilities but also to identify patients needing next-step evaluation and treatment. Thanks to the hierarchical order of all the targets assessed in each area of GAIRS, this Checklist can be a useful instrument not only for assessment but also for any eventual intervention.

## Methods

### Patient characteristics

GAIRS was administered to 113 consecutively enrolled Rett patients (111 females, mean age 18.39 ± 10.19 years), from the Italian Rett Association. All 113 patients, aged from 4 to 45 years, met the diagnostic clinical criteria for Rett Syndrome and underwent specific genetic tests.

RTT patients were classified as clinical stage III (characterized by prominent hand apraxia/dyspraxia, apparently preserved ambulation ability, and some communicative ability, mainly eye contact) or stage IV (late motor deterioration, with progressive loss of ambulation ability), according to the criteria for classic RTT by Hagberg et al. [4]. Their demographic, developmental, clinical, behavioral, and genetic information, collected from all available sources (parent/caregiver reports of history and current behavior and features, previous clinical reports, and direct observation and examination of the patients) was entered into a database.

The Mecp2 mutation was seen in 80% of the sample; specific mutations of the Mecp2 gene were: 5% showed C7D3C, 10% showed R294X, 10% showed C965C, 10% showed R255X, 15% showed P152R and 30% showed T158M. Instead, for 20%, it was not possible to specify the type of gene mutation, but all the typical phenotypic characteristics of RTT were observed. We asked the reference neuropsychiatry of each patient to give a medical judgment of severity based on typical characteristics of the syndrome (epilepsy, mood swings, convulsions, aerophagia, scoliosis). The severity level ranges from 5 (mild severity) to 20 (severe severity). Mean severity index in relation to the typical characteristics of the syndrome is 9. Table 1 shows the clinical characteristics of the participants.

**Table 1:** Clinical characteristics

	Rett patients (n = 113)
Man age (years) ± SD	18.39 ± 10.19
Range	4–45
Gender f/m	111/2
Diagnostic criteria met for Rett	113
Genetic mutations	Mecp2
Mean severity index in relation to typical characteristics of syndrome	9 (min 5–max 20)

## Materials

GAIRS is a global assessment and intervention rating scales checklist for Rett syndrome with items coming from the items of assessment in multi-disability disorders adapted to Rett syndrome [24–28]. Through a global analysis, it gives an overview of the different areas and is intended for use in a functional analysis of the overall abilities of the patient.

The GAIRS Checklist is composed of 10 macro-areas: basic or pre-requisite behavior, neuropsychological abilities, basic cognitive concepts, advanced cognitive concepts, communication abilities, emotional- affective abilities, hand motor skills, graphomotor skills, global motor abilities and level of autonomy in daily life. The 10 areas are described in Table 2. For each area, different sequential skills, hierarchically structured, are evaluated. Eighty-five skills in total are evaluated. Each skill has a numerical score ranging from 1 to 5, where 1 is the minimum level of capacity and 5 is the maximum level of capacity to perform a specific activity. Below, we present some examples. In the area of basic behavior, the first skill that is evaluated is *spontaneous eye contact*. The score of this skill is: 1 If the child is unable to establish spontaneous eye contact, 2 if the child can establish spontaneous eye contact 2/3 times out of 10, 3 if the child can establish spontaneous eye contact 4/6 times out of 10, 4 if the child can establish spontaneous eye contact 7/8 times out of 10, 5 if the child always establishes spontaneous eye contact. Instead, the sixth skill investigated in the hand motor area is *grasping ability* and its score is: 1 if the child cannot grasp an object on the table, 2 if the child can grasp a 5 cm object with palmar cubitus grip, 3 if the child can grasp a 5 cm object with palmar grip, 4 if the child can grasp a 1 cm object with pluri-digital grip, 5 if the child can grasp a 1 cm object with plier’s grip (thumb-index).

**Table 2:** Description of GAIRS checklist areas

1. Basic behaviors area Evaluates the prerequisite behaviors for learning and communication, they are: spontaneous eye contact, eye contact on request, looking at objects, tracking objects and faces, functional gestures, cooperation with simple spoken requests (reply to their name, look for mother), sitting long enough to complete a task, object permanence, be able to wait for their turn before starting an activity, be able to communicate basic needs (need to eat, drink, sleep, play, walk, go to the bathroom, and feel good or bad)	
2. Neuropsychological area Evaluates brain-based skills which are needed in acquisition of knowledge, manipulation of information, and reasoning. They have more to do with the mechanisms of how people learn, remember, problem-solve, and pay attention, rather than with actual knowledge. This area includes selective attention, types and intensity of stereotypes, lateralization, temporal orientation, spatial orientation, memory span, logical sequences, categorization (animals, dress, foods, drinks, objects, places, actions)	
3. Basic cognitive area Evaluates the basic cognitive concepts that allow the understanding of reality (spatial concepts, topological concepts, etc.). This area includes object recognition, color discrimination, geometric form discrimination measure concepts, spatial concepts, human body discriminations, time concepts, cause-effect relationship	
4. Advanced cognitive area Evaluates the concepts of school learning that include the sub-areas of writing and mathematics. This area includes global words recognition, syllables recognition, recompleting words through syllables, alphabetic symbols recognition, recompleting words with alphabetic symbols, recognition of words representing actions, using words to communicate, math pre-requisite concepts, recognition of numbers, biunivocal relation between number and quantity	
5. Communication area Evaluates the development of language by measuring responses to environmental sounds and speech, as well as the production of sounds and words. The skills of communication, comprehension and expression that allow the person to interact with others. This area includes expressing a basic need at a corporal level, recognizing, and expressing a basic need through pictures, recognizing and expressing a basic need through word, understanding the biunivocal relation of a basic need between a picture and the word that expresses it, verbal comprehension, verbal production	
6. Emotional area Evaluates the person’s abilities and ways of experiencing, expressing, and understanding their own emotions and those of others are analyzed. This area includes identify emotions and express emotions	
7. Hand motor area Evaluates the ability to make movements using the small muscles in our hands and wrists. Kids rely on these skills to do key tasks in school and in everyday life. Fine motor skills are complex, however. They involve the coordinated efforts of the brain and muscles, and they are built on the gross motor skills that allow us to make bigger movements. This area includes musculoskeletal alterations, hand–eye coordination during motor tasks lateralization, reaching movement, touching ability, grasping ability, releasing movement, repositioning movement, bimanual coordination, ability to push and pull an object	
8. Graphomotor area Evaluates the fine motor skills incorporating, among others, graphomotor skills (GS) which, in turn, involve strength and control of the finger muscles, and incorporates important daily skills such as writing and drawing, necessary for the academic achievement of all students. This area includes grasping of pencil, drawing patterns and use of school tools	
9. Global motor area Evaluates the gross-motor skills which are important for an upright posture, walking, running, and climbing. It allows for the observation of physical weakness or disability or defects of movement. This area includes: standing, sitting, parachute reactions, rolling supine—on one side, rolling supine—prone, supine—to seated on the floor, seated on the floor—to standing, seated on a chair—to standing, standing—to seated on the floor, standing—to seated on a chair, walking, body spatial orientation in standing, stepping, running, climbing upstairs, descending stairs, jumping, picking up an object from the ground (small ball), playing with a ball and walking on a slope	
10. Autonomy in daily life area Measures early adaptive and self-help behavior typically seen at home, as well as social behavior that develops through early adult–child interactions; therefore, this area analyses the level of autonomy in the praxis of daily life This area includes daily autonomy such as, eating, drinking, coughing or difficulty breathing during meal, type of food’s consistence, washing, autonomy in the bathroom and dressing, and other skills such as, playing and socialization skills and advanced autonomy activities	

VABS [23] is an assessment scale carried out by interviewing the parents and allow to highlight four domains: communication, daily living, socialization and motor skills. The reliability of the scales was established with reference to skewness, kurtosis and alpha values. Skewness is a measure of the asymmetry, kurtosis is a measure of the combined weight of a distribution's tails relative to the center of the distribution, Cronbach’s alpha is the measure of internal consistency. With reference to communication domain the reliability was established as follows: skewness = 0.38, kurtosis = 0.34, Cronbach’s alpha = 0.89; the reliability for daily living skills was: skewness = 0.34, kurtosis = 0.27, Cronbach’s alpha = 0.88; the reliability for socialization domain was: skewness = 0.69, kurtosis = 0.76, Cronbach’s alpha = 0.91; the reliability for motor skills domain was: skewness = 0.87, kurtosis = 0.67, Cronbach’s alpha = 0.94. The Cronbach’s alpha for the general scale was 0.89.

RARS [22] is a standardized scale used to evaluate the severity of the disease in patients with RTT. The total score allows to measure the severity of the disease along a continuum ranging from mild symptoms to severe ones. Skewness and kurtosis values, calculated for the distribution of the total score, are respectively 0.110 and 0.352. Distribution is found to be normal. Cronbach’s alpha is used to determine the internal consistency for the whole scale and subscales. Total alpha is 0.912, and the internal consistency of the sub-scales is high (from 0.811 to 0.934).

### Procedure

Initially, Airett center professionals contacted the family by phone through a brief interview to collect their availability for GAIRS administration sessions. Then, parents were invited to a session in which they completed the RARS scale [22] that allows to identify the severity of the patients with Rett syndrome, and the Vineland questions [23] to identify behavioral features.

After these sessions, GAIRS Checklist was administered to the patients by the Airett team, composed of a physician, speech therapist and psychologist, during the evaluation sessions at the Rett Center. All professionals had certified, special training on Rett syndrome. Total administration time was around 4 h (range from 3 to 7) but for the most serious patients it was necessary to divide the administration into multiple sessions (2 or 3). Some skill scores that cannot be given directly during the evaluation, such as the item related to the ability to go to the bathroom, was evaluated through video or interview with parents. Every skill was requested ten times, but if the participant gave the first 3 correct answers, the skill was considered acquired; in the same way, if the participant gave the first 3 wrong answers, the skill was considered not acquired.

## Results

Data were analyzed using the Statistical Package for the Social Sciences [29]. We used mean and standard deviation (SD) for the descriptive variables. Normality of the distributions of quantitative variables was verified by applying the Shapiro–Wilk test. Descriptive analysis of both demographic and clinical characteristics of Rett Syndrome patients was performed on the whole cohort. Results were discussed initially with reference to factorial structure of the GAIRS, afterwards, with reference to the internal reliability, and to the correlation for concurrent and divergent validity and finally, with reference to two examples of operational application of GAIRS and suggestions of intervention to two patients.

### Factorial structure

To verify the factorial structure of the GAIRS, exploratory factor analysis (EFA) using principal axis factoring (PAF) was conducted with the Kaiser normalization promax rotation. Finally, we considered Internal Reliability (Cronbach alpha), test–retest reliability, and Correlations for Concurrent and Divergent Validity (Pearson correlations). We considered a two-tailed p value of 0.05 or less statistically significant.

### Descriptive analyses and preliminary analyses

Tables 3, 4, 5, 6, 7, 8, 9, 10, 11 and 12 show the means and standard deviations for all the items for each subscale of GAIRS. Skewness and kurtosis for all the items were also determined and most items showed normal distribution, while few were positively or negatively skewed.

**Table 3:** Means, standard deviations, correct percentages and skewness and kurtosis values of the basic behavior area items

Basic behaviors area	Mean	Standard deviation	%	Skewness	Kurtosis
Spontaneous eye contact	3.71	1.258	74.2	− .333	− 1.349
Eye contact on request	3.59	1.256	71.8	− .208	− 1.304
Looking at objects	3.31	1.195	66.2	.136	− 1.048
Tracking objects and faces	3.27	1.262	65.4	.027	− 1.089
Functional gestures	2.24	1.129	44.8	.757	− .093
Cooperation with simple spoken requests	3.00	1.015	60.0	.355	− .363
Sitting long enough to complete a task	3.76	1.074	75.2	− .203	− 1.281
Object permanence	2.10	1.299	42.0	1.166	.297
Be able to wait for their turn before starting an activity	2.35	1.038	47.0	.632	.257
Be able to communicate basic needs	2.88	1.028	57.6	.416	− .457
Total score	3.021	.9056	60.4	.091	− .795

**Table 4:** Means, standard deviations, correct percentages and skewness and kurtosis values of the neuropsychological area items

Neuropsychological area	Mean	Standard deviation	%	Skewness	Kurtosis
Types and intensity of stereotypes	2.67	.965	53.4	.361	− .290
Lateralization	2.71	1.085	54.2	.158	− .290
Temporal orientation	1.53	.979	30.6	.314	− .290
Spatial orientation	1.45	1.009	29.0	1.858	− .290
Memory span	1.52	.870	30.4	2.127	− .290
Logical sequences	1.23	.777	24.6	1.580	− .290
Categorization	1.18	.730	23.6	3.270	− .290
Total score	1.88	.7126	37.6	2.118	5.593

**Table 5:** Means, standard deviations, correct percentages and skewness and kurtosis values of the basic cognitive area items

Basic cognitive area	Mean	Standard deviation	%	Skewness	Kurtosis
Object recognition	3.43	1.174	68.6	.057	− 1.079
Color discrimination	2.65	1.351	53.0	.364	− .966
Geometric form discrimination	2.21	1.387	44.2	.818	− .480
Measure concepts	1.78	1.124	35.6	1.709	2.553
Spatial concepts	1.29	.844	25.8	3.204	2.450
Human body discriminations	2.03	1.226	40.6	.981	.185
Time concepts	1.33	.943	26.6	2.973	8.404
Cause-effect relationship	1.19	.720	23.8	3.997	2.450
Total score	1.988	.93	39.8	1.410	1.845

**Table 6:** Means, standard deviations, correct percentages and skewness and kurtosis values of the advanced cognitive area items

Advanced cognitive area	Mean	Standard deviation	%	Skewness	Kurtosis
Global words recognition	1.41	.922	28.2	2.478	6.227
Syllable’s recognition	1.16	.721	23.2	4.703	23.010
Recompleting words through syllables	1.14	.725	22.8	4.810	23.328
Alphabetic symbols recognition	1.16	.801	23.2	4.524	19.700
Recompleting words with alphabetic symbols	1.15	.687	23.0	4.562	21.956
Recognition of words representing actions	1.14	.752	22.8	4.700	21.512
Using words to communicate	1.19	.662	23.2	3.608	14.800
Math pre-requisite concepts	1.52	.858	30.4	1.794	3.492
Recognition of numbers	1.57	.946	31.4	1.916	3.992
Biunivocal relation between number and quantity	1.57	1.305	31.4	4.439	27.658
Total score	1.30	.6938	26.0	3.620	15.089

**Table 7:** Means, standard deviations, correct percentages and skewness and kurtosis values of the communication area items

Communication area	Mean	Standard deviation	%	Skewness	Kurtosis
Expressing a basic need at a physical level	2.64	1.197	52.8	.224	− .715
Recognizing and expressing a basic need through pictures	2.33	1.250	46.0	.577	− .562
Understanding the biunivocal relation between the corpora	2.24	1.274	44.8	.725	− .395
Recognizing and expressing a basic need through word	1.34	.952	26.8	2.755	6.876
Understanding the biunivocal relation of a basic need between a picture and the word that expresses it	1.24	.789	24.8	3.408	11.969
Verbal comprehension	2.83	1.068	56.6	− .058	− .183
Verbal production	1.42	.840	28.4	2.183	4.954
Total score	2.00	.80343	40.1	1.123	1.550

**Table 8:** Means, standard deviations, correct percentages and skewness and kurtosis values of the emotional area items

Emotional area	Mean	Standard deviation	%	Skewness	Kurtosis
Identify emotions	2.28	1.218	45.6	.636	− .317
Express emotions	3.04	1.038	60.8	− .026	− .468
Total score	2.66	1.019	53.2	.505	− .210

**Table 9:** Means, standard deviations, correct percentages and skewness and kurtosis values of the hand motor area items

Hand motor area	Mean	Standard deviation	%	Skewness	Kurtosis
Musculoskeletal alterations	3.06	1.286	61.2	.323	− .404
Hand–eye coordination during motor tasks	2.80	1.223	56.0	.562	.326
Lateralization	2.76	1.190	55.2	.442	− .597
Reaching movement	2.86	1.349	57.2	.386	− .418
Touching ability	2.86	1.429	57.2	.336	− .698
Grasping ability	2.30	1.360	46.0	.593	− .973
Releasing movement	1.95	1.359	39.0	1.054	− .285
Repositioning movement	1.79	1.217	35.8	1.237	.342
Bimanual coordination	1.88	1.122	37.6	1.118	.464
Ability to push and pull an object	2.00	1.363	40.0	1.196	.126
Total score	2.43	1.078	48.6	− .752	2.426

**Table 10:** Means, standard deviations, correct percentages and skewness and kurtosis values of the graphomotor area items

Graphomotor area	Mean	Standard deviation	%	Skewness	Kurtosis
Grasping of pencil	1.91	1.083	38.2	1.009	.087
Drawing patterns	1.72	1.016	34.4	1.475	1.516
Use of school tools	1.41	.842	28.2	2.620	7.251
Total score	1.68	.89508	33.6	1.560	2.341

**Table 11:** Means, standard deviations, correct percentages and skewness and kurtosis values of the gross motor area items

Gross motor area	Mean	Standard deviation	%	Skewness	Kurtosis
Standing	3.21	1.358	64.2	− .070	− 1.286
Sitting	3.37	1.376	67.4	− .222	− 1.270
Parachute reactions	2.74	1.169	54.8	.563	− .485
Rolling supine—on one side	2.67	1.035	53.4	.368	− .376
Rolling supine—prone	2.61	1.024	52.2	.330	− .363
Supine—to seated on the floor	2.80	2.184	56.0	6.974	61.201
Seated on the floor—to standing	2.55	.957	51.0	.313	− .072
Seated on a chair—to standing	2.59	.965	51.8	.221	− .162
Standing—to seated on the floor	2.52	.979	50.4	.305	− .168
Standing—to seated on a chair	2.56	.988	51.2	.215	− .262
Walking	3.09	1.288	61.8	.090	− 1.149
Body spatial orientation in standing	2.49	1.193	49.8	.606	− .362
Stepping	2.48	1.078	49.6	.572	− .255
Running	1.94	1.171	38.8	.890	− .359
Climbing upstairs	2.36	.969	47.2	.304	− .258
Descending stairs	2.38	.940	47.6	.356	− .069
Jumping	1.23	.664	24.6	3.289	13.242
Picking up an object from the ground (small ball)	1.31	.873	26.2	3.070	9.551
Playing with a ball	1.67	.911	33.4	1.527	2.802
Walking on a slope	2.34	1.085	46.8	.689	− .006
Total score	2.46	.88757	49.2	.505	.055

**Table 12:** Means, standard deviations, correct percentages and skewness and kurtosis values of the autonomy in daily life area

Autonomy in daily life area	Mean	Standard deviation	%	Skewness	Kurtosis
Eating	2.57	.913	51.4	.886	.407
Drinking	2.50	.870	50.6	1.079	1.156
Coughing or difficulty breathing during meal	2.83	1.016	56.6	.114	− .681
Type of textures he usually eats	3.08	1.061	61.6	.304	− .748
Washing	2.20	.791	44.0	.624	.928
Autonomy in the bathroom	2.07	.795	41.4	.736	1.190
Dressing	2.11	.695	42.2	.218	− .035
Playing area	2.13	.812	42.6	.218	− .566
Socialization area	2.75	.903	55.0	− .068	− .521
Advanced autonomy area	1.17	.496	23.0	3.428	3.450
Total score	2.28	.70815	45.6	1.125	1.857

Inter-subscale correlations were very high (Table 13). All 10 subscales of GAIRS were positively and significantly related to each other and to the total GAIRS score.

**Table 13:** Inter-subscale correlations among GAIRS’ areas

GAIRS Checklist areas	1	2	3	4	5	6	7	8	9	10	11
(1) Basic behaviors area	1										
(2) Neuropsychological area	.739**	1									
(3) Basic cognitive area	.653**	.824**	1								
(4) Advanced cognitive area	.435**	.663**	.712**	1							
(5) Communication area	.699**	.787**	.814**	.710**	1						
(6) Emotional area	.626**	.694**	.802**	.643**	.815**	1					
(7) Hand motor area	.689**	.538**	.430**	.221*	.524**	.497**	1				
(8) Graphomotor area	.568**	.635**	.512**	.382**	.577**	.517**	.834**	1			
(9) Gross motor area	.533**	.580**	.488**	.364**	.572**	.511**	.756**	.730**	1		
(10) Autonomy area	.692**	.662**	.561**	.436**	.658**	.591**	.778**	.714**	.859**	1	
Total score	.821**	.844**	.783**	.625**	.838**	.765**	.820**	.805**	.860**	.892**	1

*p < 0.05; **p < 0.01

### Exploratory factor analysis

To verify the factorial structure of GAIRS, PAF was conducted with the Kaiser normalization promax rotation. The use of an EFA approach in a first study testing a new construct such as GAIRS is suitable [30]. Furthermore, the use of PAF is recommended with a violation of the assumption of multivariate normality [31, 32].

The number of factors was determined through Velicer’s minimum average partial (MAP) test and parallel analysis [33]. Both the parallel analysis and the original MAP test suggested two factors, and for this reason, a PAF estimation using promax rotation with two-factor solutions was used to explore factor loadings. The two-factor solution was found to explain 60% of the variance. Almost all the items are loaded into the first factor (that explains 40% of variance) and provide evidence of a general level of abilities which are homogeneous in all subareas. The second factor explains 20% of the variance and can provide evidence of advanced formal learning abilities (Table 14).

**Table 14:** Exploratory factor analysis

GAIRS skills	Components 1	Components 2
Spontaneous eye contact	**.599**	− .128
Eye contact on request	**.655**	− .150
Looking at objects	**.705**	− .025
Tracking objects and faces	**.707**	− .052
Functional gestures	**.752**	− .090
Cooperation with simple spoken requests	**.631**	.085
Sitting long enough to complete a task	**.568**	− .140
Object permanence	**.637**	.282
Be able to wait for their turn before starting an activity	**.700**	.266
Be able to communicate basic needs	**.599**	− .128
Selective attention	**.669**	.237
Types and intensity of stereotypes	**.452**	.123
Lateralization	**.571**	.123
Temporal orientation	**.745**	.269
Spatial orientation	**.746**	.340
Memory span	**.700**	.388
Logical sequences	**.683**	.318
Categorization	**.706**	.390
Object recognition	**.638**	.166
Color discrimination	**.641**	.335
Geometric form discrimination	**.640**	.406
Measure concepts	**.612**	.479
Spatial concepts	**.679**	.474
Human body discriminations	**.715**	.474
Time concepts	**.727**	.456
Cause-effect relationship	**.732**	.401
Expressing a basic need at a corporal level	**.618**	.177
Recognizing and expressing a basic need through pictures	**.639**	.291
Understanding the biunivocal relation between the corpora	**.652**	.337
Recognizing and expressing a basic need through word	**.674**	.386
Understanding the biunivocal relation of a basic need between a picture and the word	**.577**	.485
Verbal comprehension	**.712**	.257
Verbal production	**.605**	− .105
Identify emotions	**.645**	.454
Express emotions	**.733**	.059
Musculoskeletal alterations	**.512**	− .584
Hand–eye coordination during motor tasks	**.594**	− .490
Lateralization	**.605**	− .338
Reaching movement	**.718**	− .456
Touching ability	**.697**	− .434
Grasping ability	**.730**	− .380
Releasing movement	**.729**	− .262
Repositioning movement	**.717**	− .223
Bimanual coordination	**.774**	− .177
Ability to push and pull an object	**.707**	− .333
Grasping of pencil	**.760**	− .290
Drawing patterns	**.682**	− .274
Use of school tools	**.789**	.037
Standing	**.685**	− .317
Sitting	**.672**	− .410
Parachute reactions	**.724**	− .417
Rolling supine—on one side	**.743**	− .401
Rolling supine—prone	**.764**	− .336
Supine—to seated on the floor	**.799**	− .332
Seated on the floor—to standing	**.799**	− .361
Seated on a chair—to standing	**.781**	− .321
Standing—to seated on the floor	**.785**	− .336
Standing—to seated on a chair	**.712**	− .391
Walking	**.726**	− .318
Body spatial orientation in standing	**.733**	− .315
Stepping	**.698**	− .269
Running	**.766**	− .270
Climbing upstairs	**.768**	− .257
Descending stairs	**.545**	.096
Jumping	**.705**	− .007
Picking up an object from the ground (small ball)	**.766**	− .186
Playing with a ball	**.685**	− .475
Walking on a slope	**.685**	− .317
Eating	**.804**	− .130
Drinking	**.816**	− .129
Coughing or difficulty breathing during meal	**.629**	− .150
Type of textures he usually eats	**.686**	− .268
Washing	**.714**	− .145
Dressing	**.677**	− .134
Playing area	**.600**	− .235
Socialization area	**.676**	− .264
Advanced autonomy area	**.610**	− .236
Global words recognition	.564	**.627**
Syllables recognition	.473	**.615**
Recompleting words through syllables	.509	**.599**
Alphabetic symbols recognition	.540	**.575**
Recompleting words with alphabetic symbols	.557	**.600**
Recognition of words representing actions	.537	**.576**
Using words to communicate	.574	**.644**
Math pre-requisite concepts	.523	**.563**
Recognition of numbers	.588	**.601**
Biunivocal relation between number and quantity	.564	**.627**

The first part of satured items which belong to the first factors has to be in bold, and the second part of the items which belong to the second factor has to be in bold

### Internal reliability, and correlation for concurrent and divergent validity

Since we obtained a single general factor explaining 40% of the variance of GAIRS, we decided to maintain the original sub areas to better describe the general functioning of patients with Rett Syndrome in each of them. For each subarea, we calculated the alpha levels for internal consistency. Cronbach’s alpha values for all the subscales and the total score were high with a range from 0.77 to 0.95. A test–retest correlation was applied on 58 patients with Rett syndrome 2 months after the first administration and the results were very high (r (58) = 0.82, p < 0.002).

All ten subscales of GAIRS were negatively related with the severity of symptoms of the RARS scale [22] and with neuropsychiatric symptom evaluations, while all the subscales of GAIRS were positively correlated with the concurrent measure of the Vineland (Table 15).

**Table 15:** Correlation among GAIRS score and other assessment instruments

GAIRS area	Neuropsychiatric evaluation	RARS score	Vineland score
Basic behaviors area	− .492**	− .428**	.651**
Neuropsychological area	− .440**	− .528**	.628**
Basic cognitive area	− .377**	− .510**	.647**
Advanced cognitive area	− .437**	− .523**	.374*
Communication area	− .441**	− .377**	.680**
Emotional area	− .493**	− .323*	.586**
Hand motor area	− .437**	− .289*	.693**
Graphomotor area	− .424**	− .269	.677**
Gross motor area	− .416**	− .327*	.729**
Autonomy in daily life area	− .434**	− .476**	.687**
Total score	− .488**	− .486**	.726**

**p < .01

*p < .05

### Applications of GAIRS and suggestions of intervention

GAIRS scores have a mean of 2.28 and a standard deviation of 0.70. Based on this, we divided the scores on three levels: patient scores that fall in the 1–1.58 range (where the maximum is m-sd, i.e., 2.28–0.70) show basic competence, patient scores that fall in the 1.59–2.98 range (where the maximum is m + sd, i.e. 2.28 + 0.70) show an intermediate level of competence and, finally, patient scores that that fall in the 2.99–5.00 range show a high level of competence.

**Fig. 1:**
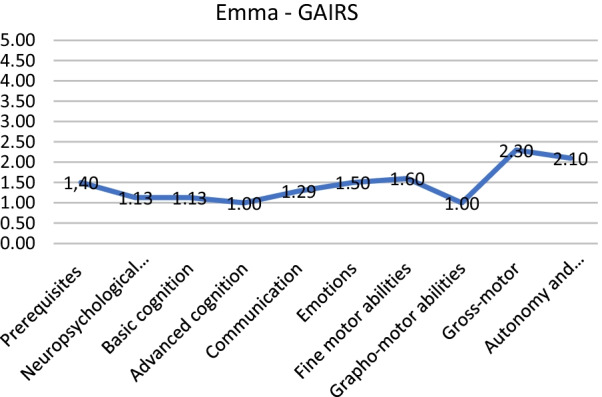
GAIRS mean scores for each area

**Fig. 2:**
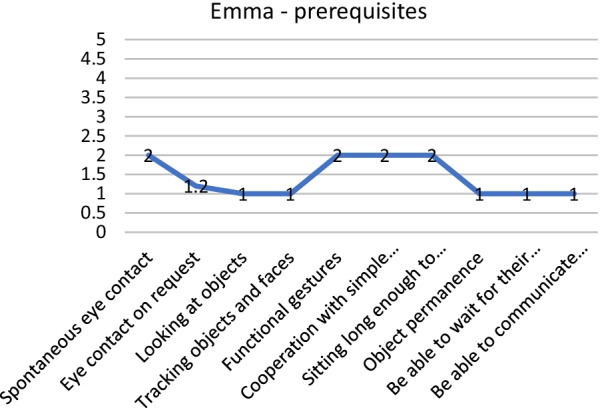
Prerequisites subarea of GAIRS mean scores

As an example, below, we present two patients (from our data file) that benefitted from the administration of GAIRS. Emma (name changed for anonymity) has a general score of 1.56, this means that she has a basic level of competence. This index told us nothing about intervention. We need to examine all areas of GAIRS: as we can see from Fig. 1, Emma has an intermediate level of gross motor abilities and participation and autonomy. She needs to improve prerequisites. For this reason, it is useful to go more in depth and to disaggregate prerequisite data, as shown in Fig. 2. We can see that while sometimes she looks for people with spontaneous eye contact, she is not able to show eye contact on request, neither look nor trace an object. Then we continue by examining the other areas. Once the hypothesis for each patient was developed, differential reinforcement procedures combined with extinction were designed in order to increase the identified behaviors. For example, the training with Emma has to have the aim of reaching a higher level of visual attention to faces and to objects. Additionally, she may benefit from starting functional communication training and motor training.

**Fig. 3:**
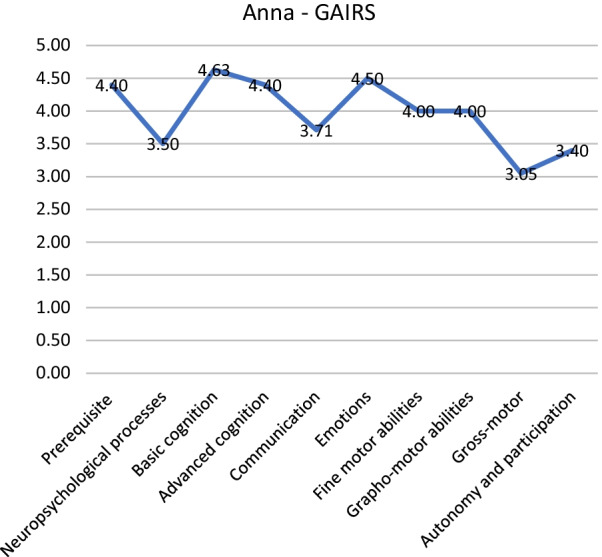
GAIRS mean scores for each area

**Fig. 4:**
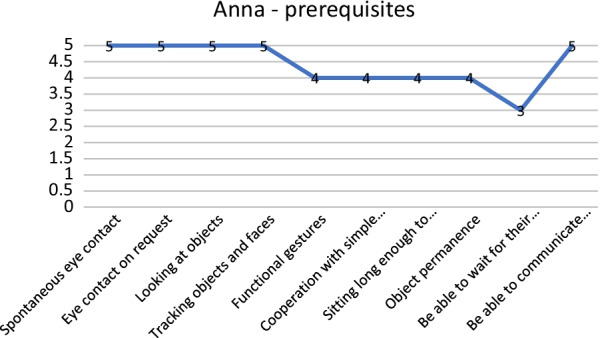
Prerequisites subarea of GAIRS mean scores

Another example of application is the analysis of GAIRS with Anna. Anna (name changed) has a general score of 3.90, this means that she has a high level of competence. Again, we have to examine all areas of GAIRS: as we can see from Fig. 3, Anna has almost a master level in the prerequisites area of abilities and participation and autonomy. The disaggregation of prerequisites shows that she does not need to improve prerequisites (Fig. 4), just the ability to wait. We can continue and disaggregate the other areas and produce the hypothesis for the intervention.

GAIRS scores have a mean of 2.28 and a standard deviation of 0.70. Based on this, we divided the scores on three levels: patient scores that fall in the 1–1.58 range (where the maximum is m-sd, i.e., 2.28–0.70) show basic competence, patient scores that fall in the 1.59–2.98 range (where the maximum is m + sd, i.e. 2.28 + 0.70) show an intermediate level of competence and, finally, patient scores that that fall in the 2.99–5.00 range show a high level of competence.

## Discussion

The principal aim of this study was to examine the psychometric characteristics of the GAIRS Checklist. Moreover, the aim was to examine the validity of GAIRS with test–retest correlation, convergent validity with similar functional measurements such as the Vineland scales [23], divergent validity with severity of disease scale such as the RARS [22] scale and neuropsychiatric evaluations.

With reference to the validity of GAIRS, we decided to maintain the original subareas of the Checklist to better describe the general functioning of patients with Rett Syndrome in each of them. For these reasons, this study adds data on the use of the GAIRS Checklist in the global evaluation of patients with RTT. The results of the statistical analysis showed good internal reliability of the scale. Regarding the convergent validity with similar functional measurements such as the Vineland scales, all subscales of GAIRS were positively correlated with the concurrent measure of the Vineland and with neuropsychiatric symptom evaluation. With reference to the severity of disease scale such as the RARS scale and the neuropsychiatric evaluations, all ten sub-scales of GAIRS were negatively related with the severity of symptoms of the RARS scale [22]. All subscales of GAIRS were positively correlated with the concurrent measure of the Vineland and with neuropsychiatric symptom evaluation.

Our experience confirms the previously reported findings and suggests that the GAIRS Checklist can be used to assess behavioral, intellectual, academic, neuropsychological and psychosocial manifestations in patients with Rett Syndrome. The use of this Checklist can be extended to screen for neuropsychiatric involvement in RTT with complex needs. It can be integrated into routine medical appointments of individuals and conducted by all the therapists involved in the assessment and intervention with RTT patients. If the aim of the intervention is more related to motor abilities of the girls with Rett syndrome the GAIRS can be integrated with motor scales [19, 20].

Furthermore, the checklist can be easily re-administered during follow-up to detect behavioral and psychological changes over time and the efficacy of therapeutic intervention.

These results have interesting implications for future rehabilitation for deeply impaired clinical conditions as in the case of RTT [34–37]. First of all, this study may indicate a way to possibly modify RTT patients’ cognitive, motor and communicational structure, and improve their quality of life, as well as the quality of life in the people close to them.

GAIRS indeed is a descriptive assessment that involves completing direct observations in different environments and recording data as behaviors occur in order to determine the maintaining function of a behavior. It provides a structure for assimilation and integration of information leading to mastery of effective clinical reasoning in occupational therapy assessment and intervention. The model adheres to the World Health Organization in the International Classification of Functioning, Disability and Health (ICF) [38], in which both individual and environmental factors that enable or constrain participation in the community are considered in relation to health. The theoretical Model used here is the Applied Behavior Analysis (ABA). ABA is the science in which tactics derived from the principles of behavior are applied systematically to improve socially significant behaviors and experimentation is used to identify the variables responsible for behavior change [39]. ABA methods serve to identify the cause of a behavior and seek ways to improve behavior based on the identified function.

Despite the satisfactory psychometric characteristics, extension studies are warranted, also involving training in order to fully characterize the long-term evolution of GAIRS in Rett syndrome. Lastly, investigating the role intensive and low-frequency trainings on modifying GAIRS areas could provide further evidence about the ability of GAIRS checklist to discriminate between different clinical samples.

## Conclusion

The GAIRS checklist was developed to provide healthcare professionals with a tool to easily screen neuro-psychiatric involvement in patients with RTT. The checklist explores the frequency of a wide range of neuropsychiatric manifestations and multiple dimensions of involvement on different levels: behavioral, psychiatric, intellectual, academic, neuropsychological and psychosocial. As these aspects can be impaired in RTT patients, we hypothesized that the checklist could be useful for screening neuropsychiatric needs in this population and for suggesting rehabilitation interventions.

## Data Availability

The data can be obtained from the corresponding author upon request.

## Abbreviations

RTT: Rett syndrome
GAIRS: Global Assessment and Intervention in Rett Syndrome
VABS: Vineland Adaptive Behavior Scale Interview
RARS: Rett Assessment Rating Scale
RSGMS: Rett Syndrome Gross Motor Scale
RESMES: Rett Syndrome Motor Evaluation Scale
AIRETT: Italian Rett Association
